# Highly Diverse Symbiodiniaceae Types Hosted by Corals in a Global Hotspot of Marine Biodiversity

**DOI:** 10.1007/s00248-024-02407-x

**Published:** 2024-07-10

**Authors:** Ming Sheng Ng, Nathaniel Soon, Lutfi Afiq-Rosli, Ismael Kunning, Ralph R. Mana, Ying Chang, Benjamin J. Wainwright

**Affiliations:** 1 Department of Biological Sciences, National University of Singapore, Singapore, Singapore; 2 Yale-NUS College, National University of Singapore, 16 College Avenue West, Singapore, 138527 Singapore; 3 Thrive Conservation, Jl. Subak Sari No. 13, Kuta Utara, Badung, Bali, 80361 Indonesia; 4 Tropical Marine Science Institute, National University of Singapore, Singapore, Singapore; 5 Red Sea Research Center, Biological and Environmental Sciences and Engineering Division (BESE), King Abdullah University of Science and Technology (KAUST), Thuwal, Saudi Arabia; 6 School of Natural and Physical Sciences, University of Papua New Guinea, Port Moresby, Papua New Guinea

**Keywords:** Papua New Guinea, Coral Triangle, Oceania, SymPortal, Symbiodiniaceae, High-throughput sequencing

## Abstract

Symbiotic dinoflagellates in the genus Symbiodiniaceae play vital roles in promoting resilience and increasing stress tolerance in their coral hosts. While much of the world’s coral succumb to the stresses associated with increasingly severe and frequent thermal bleaching events, live coral cover in Papua New Guinea (PNG) remains some of the highest reported globally despite the historically warm waters surrounding the country. Yet, in spite of the high coral cover in PNG and the acknowledged roles Symbiodiniaceae play within their hosts, these communities have not been characterized in this global biodiversity hotspot. Using high-throughput sequencing of the ITS2 rDNA gene, we profiled the endosymbionts of four coral species, *Diploastrea heliopora*, *Pachyseris speciosa*, *Pocillopora acuta*, and *Porites lutea*, across six sites in PNG. Our findings reveal patterns of *Cladocopium* and *Durusdinium* dominance similar to other reefs in the Coral Triangle, albeit with much greater intra- and intergenomic variation. Host- and site-specific variations in Symbiodiniaceae type profiles were observed across collection sites, appearing to be driven by environmental conditions. Notably, the extensive intra- and intergenomic variation, coupled with many previously unreported sequences, highlight PNG as a potential hotspot of symbiont diversity. This work represents the first characterization of the coral-symbiont community structure in the PNG marine biodiversity hotspot, serving as a baseline for future studies.

**Supplementary Information:**

The online version contains supplementary material available at 10.1007/s00248-024-02407-x.

## Introduction

Papua New Guinea, located on the eastern edge of the biologically diverse Coral Triangle [1], is a global marine biodiversity hotspot with over 500 coral and 860 fish species across more than 600 islands [2–4]. Embedded within the West Pacific Warm Pool, PNG is characterized by some of the warmest seawaters of the global ocean [5]. Compared to its Indo-Pacific and Australian neighbors, PNG has warmer seas with water temperatures regularly reaching 29 to 30 °C, resulting in over 10 times more thermal stress events (e.g., degree heating weeks ≥ 4) [6, 7]. Yet, live coral cover in PNG is among the highest in the world with a reported countrywide average of 50%, surpassing the global average of below 30% [8]. This resilience is a testament to the ability of corals from PNG to adapt to challenging thermal conditions and recover from past stressors such as thermally induced bleaching, crown-of-thorns starfish invasion, and sedimentation [4, 8].

However, recent overexploitation of marine resources in PNG has resulted in coral cover plummeting from 75% to less than 10% in some locations [9–11]. The hundreds of isolated islands in PNG and the associated transportation difficulties make trade inaccessible, causing an estimated 85% of the country’s nine million population to depend almost entirely on the surrounding coral reefs for food and other daily needs [8]. This reliance and decline in coral cover has reduced fish abundance by more than half, and if left unchecked, it is predicted that many other marine food sources will also be reduced [12]. Such declines will have negative ecological impacts on marine ecosystems and create hardships for the communities that rely upon them.

The survival and resilience of coral reefs and their associated communities is largely contingent on the mutualistic relationship formed between the coral hosts and their dinoflagellate endosymbionts. Originating from the highly diverse family Symbiodiniaceae (Phylum: Myzozoa), these photosynthetic endosymbionts provide the coral host up to 90% of its nutritional requirements through photosynthate translocation [13, 14]. Symbiodiniaceae also play important roles in coral skeletal calcification [15, 16] and nutrient conservation and recycling [17]. In return, the algal symbionts receive respiratory CO_2_ and nitrogenous waste products, and gain access to downwelling light and protection from grazers [18]. This endosymbiotic relationship is paramount in coral reef ecosystems, with the expulsion of symbiont during thermally induced coral bleaching, a key cause of extensive coral mortality and reef degradation globally [8].

Corals are often dominated by one or two Symbiodiniaceae species at a given time [19–21] and can adapt to warmer waters by restructuring towards heat-tolerant Symbiodiniaceae [22–25]. For instance, in response to stressors such as bleaching, corals can shuffle the proportion of their heat-tolerant symbionts [26], and/or switch to different symbionts acquired from the environment [27]. To survive the historically high sea surface temperatures of the West Pacific Warm Pool, the corals of PNG have likely adopted similar strategies to help alleviate the impact of thermal stress events. Characterizing the coral-associated Symbiodiniaceae partners is an important step in understanding how this resilience facilitates the high coral cover in PNG. Among the eleven formally described genera today [28–31], *Symbiodinium*, *Cladocopium*, and *Durusdinium* (formerly Clade A, C, D) are the most common in Indo-Pacific corals [32]. Briefly, *Symbiodinium* thrives in high light or variable light conditions [33]; *Cladocopium* is ecologically diverse, associated with various hosts [34], and often provides their juvenile coral hosts with faster growth rates than other symbionts [35, 36]; while *Durusdinium* is recognized for its tolerance to large temperature and turbidity fluctuations [37]. While it currently boasts a high coral cover, PNG is predicted to experience accelerated warming relative to the global average [6, 7], and these rising sea surface temperatures coupled with high temperature anomalies in the region will make coral bleaching in PNG more frequent and severe [38]. Without knowing how the Symbiodiniaceae communities in PNG are currently structured, it becomes impossible to assess the direction and magnitude of any future changes.

Here, we characterized the endosymbionts of four coral species, *Diploastrea heliopora*, *Pachyseris speciosa*, *Pocillopora acuta*, and *Porites lutea*, across six sites in PNG by profiling the ITS2 rDNA marker. These corals were selected because they are readily identifiable, abundant, and have contrasting life histories. In short, both *Porites lutea* and *Diploastrea heliopora* are stress-tolerant, broadcast spawners with predominantly gonochoric polyps [39–41]; *Pachyseris speciosa* is a generalist, gonochoric spawning coral [41, 42]; while *Pocillopora acuta* is a fast-growing, opportunistic, and weedy hermaphrodite with a mixed mode of reproduction [42, 43]. As the first study to characterize coral-Symbiodiniaceae communities in PNG, this work will provide important insights and serve as an important baseline for future research.

## Methods

### Coral Sampling

**Fig. 1:**
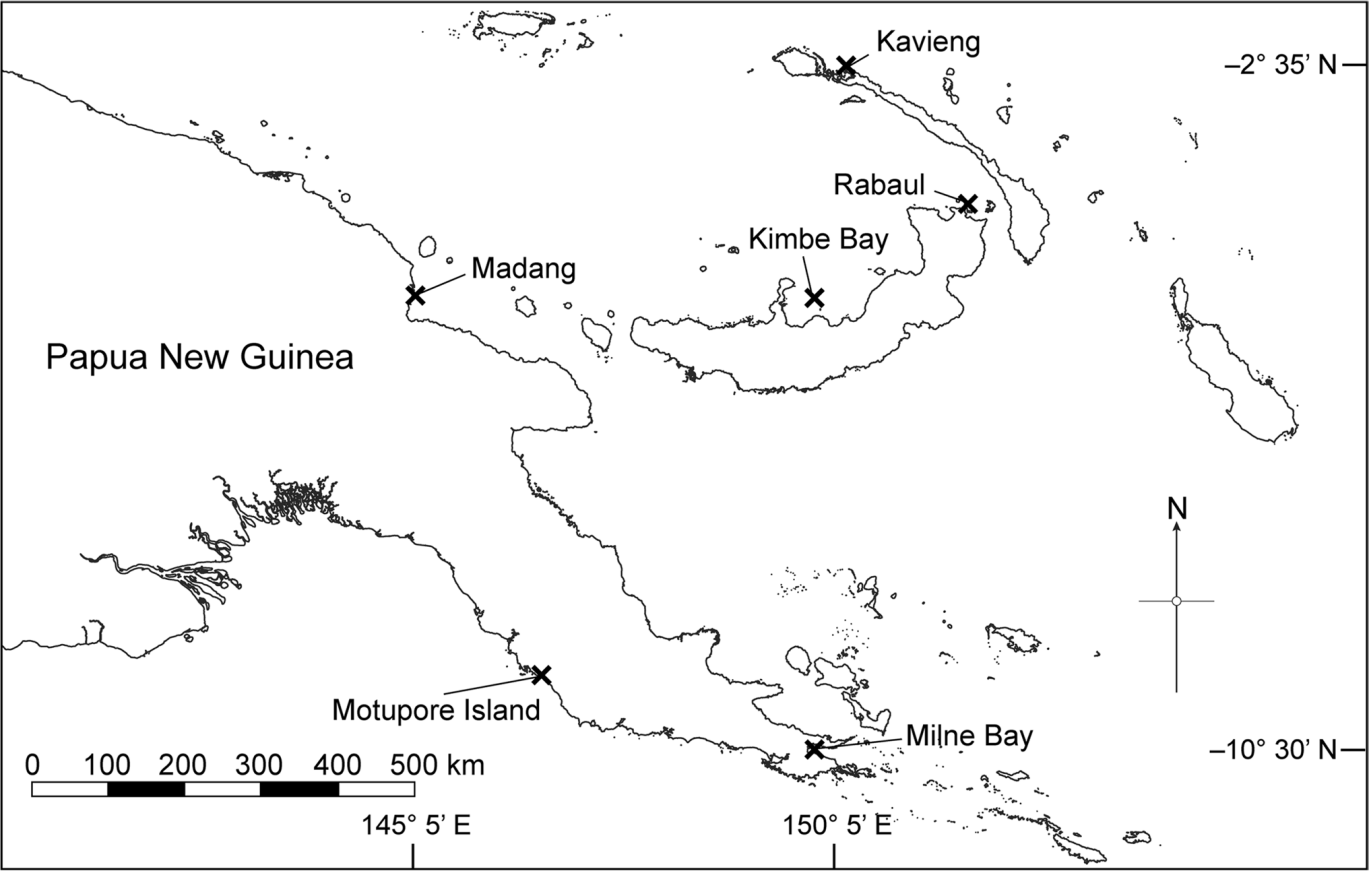
The six sampling sites of Papua New Guinea where the coral colonies were collected

Four coral species, *Diploastrea heliopora*, *Pachyseris speciosa*, *Pocillopora acuta*, and *Porites lutea*, were sampled in September 2018 from six sites across Papua New Guinea (PNG), namely, Kavieng, Rabaul, Kimbe Bay, Madang, Motupore Island, and Milne Bay (Fig. 1). All corals were collected from inshore reefs at depths between 5 and 10 m. Twenty samples per coral species were collected from each site. Samples were collected from visibly healthy individuals showing no signs of bleaching or disease. As per Wainwright et al. [44], collected samples were placed in separate sealed containers filled with seawater and kept in the shade until they could be preserved in 100% molecular-grade ethanol.

### DNA Library Preparation and Sequencing

DNA was extracted from all samples using the DNeasy Blood and Tissue Kit (Qiagen) according to the manufacturer’s protocol. We followed the library preparation of Soon et al. [45] using the SYM_VAR primer pair for Symbiodiniaceae ITS2 amplification (Table S1) [46]. Polymerase chain reaction (PCR) was performed using 1 µL of sample DNA diluted 1:10 with PCR grade water, 0.5 µL of forward and reverse primers each, 6.25 µL of 2 × KAPA HiFi HotStart ReadyMix, and water to make a final volume of 12.5 µL per reaction mix. The PCR cycling conditions began with an initial step of 95 °C for 3 min followed by 25 cycles of 95 °C for 30 s, 55 °C for 30 s, and 72 °C for 30 s, before a final extension step at 72 °C for 5 min. PCR products were purified using AMPure XP magnetic beads (Agencourt) prior to indexing. Adaptor-index PCR was then performed using 2 µL of amplicon from the previous round, 0.5 µL of each uniquely indexed primer, 6.25 µL of 2 × KAPA HiFi HotStart ReadyMix, and water to a total volume of 12.5 µL per reaction. The PCR cycling conditions were 95 °C for 3 min for initial denaturation, followed by 8 cycles of 95 °C for 30 s, 55 °C for 30 s, and 72 °C for 30 s, with a final extension step at 72 °C for 5 min (see Table S1 for full details and primer sequences).

Successful PCR amplification for each step was verified by running 1 µL of each PCR product on a 1% agarose gel. Sample normalization was performed using the SequelPrep Normalization Plate Kit (Invitrogen, Carlsbad, CA, USA) to achieve a concentration of 1–2 ng/µL per reaction. Samples were then pooled and libraries were sequenced on the Illumina MiSeq platform (V3 chemistry, 300-bp paired-end reads, 30% PhiX spike) by Macrogen Inc.

### Bioinformatics

Sequence data was processed using the SymPortal framework [47]. Demultiplexed and paired forward and reverse FASTQ files were submitted to SymPortal for sequence filtering and quality control. Contiguous sequences generated from Mothur were then screened with maximum ambiguities allowed set to 0 and maximum homopolymer set to 5 to discard reads generated from sequencing errors. Distinct sequences were identified, with singletons and doubletons excluded from further analysis. Non-Symbiodiniaceae sequences were then removed by searching against the SymPortal reference database on a sample-by-sample basis with BLASTn. Sequences were classified as Symbiodiniaceae if they shared identity > 80% and coverage > 95% to any sequence in the database using default settings. Size screening was performed with minimum and maximum cut-offs of 184 bp and 310 bp, respectively. Finally, the minimum entropy decomposition (MED) nodes were determined within a given set of sequences by the nucleotide positions most informative in distinguishing between these sequences to generate relative abundance data. SymPortal identifies and generates defining intragenomic variants (DIVs) as distinct sequences repeatedly found across multiple samples. These DIVs are subsequently used to characterize ITS2 type profiles, representing a set of co-occurring DIVs [47]. Samples containing fewer than 10,000 sequencing reads were excluded and rarefaction curves were plotted for the remaining samples to ensure that adequate sequencing depth had been attained (Fig. S1).

### Environmental Data

Environmental data associated with the collection sites was extracted from Bio-ORACLE v2.1 [48]. All data layers were from the surface at a resolution of 5 arcmin, or approximately 9.2 km at the equator, and the long-term averages of each parameter at each site were extracted from their respective layers. Fourteen parameters were first obtained as baseline environmental conditions at each collection site. These were sea surface temperature, photosynthetically active radiation (PAR), pH, salinity, cloud cover, water clarity, chlorophyll *a* concentration, current velocity, dissolved oxygen levels, primary productivity, and silicate, calcite, nitrate, and phosphate concentrations. Multicollinearity was performed with the *GGally* package in R [49] to remove parameters with pairwise collinearity values greater than 0.7, and environmental parameters with the most biological significance to coral and Symbiodiniaceae physiology were retained (e.g., parameters related to photosynthesis and thermal tolerance). Four Bio-ORACLE parameters were retained and used for subsequent analyses: mean sea surface temperature (SST) (°C), mean photosynthetically active radiation (E m^−2^ d^−1^), mean pH, and mean salinity (PSS) (Table S2). Additionally, the number of thermal stress events that were likely to cause coral bleaching between 1985 and 2023, measured by degree heating weeks that reached or exceeded 4 °C-weeks [50], were obtained from NOAA Coral Reef Watch [51] (Fig. S2, Table S3).

### Statistical Analyses

All statistical analyses were conducted in R [52]. To investigate both sequence and ecological beta diversity, we used both the sequence data and the defining intragenomic variant (DIV) compositions generated from SymPortal respectively. For sequence beta-diversity, a principal coordinate analysis (PCoA) plot with UniFrac distances was constructed to visualize the sample and type-profile clustering of *Durusdinium* and *Cladocopium* genotypes separately. Permutational analysis of variance (PERMANOVA) with 999 permutations was also conducted on the between-sample UniFrac distances to investigate how the type profiles were structured by host and site effects. To minimize spurious correlations, ecological beta-diversity analyses were conducted following the Compositional Dataset (CoDa) analysis framework [53] using the robust Aitchison distance metric [54] with the *vegdist* function from the *vegan* package [55]. A principal component analysis (PCA) plot was first constructed to visualize the Symbiodiniaceae DIV compositions hosted by the four coral species. PERMANOVA with 999 permutations was then conducted via the *adonis2* function in the *vegan* package to investigate whether DIV compositions were significantly structured by coral species and collection site, and their interaction effect. Following results showing significant differences across coral species, analyses were then conducted separately for each coral species. Compositions of DIVs were first visualized via PCA for each host species. PERMANOVA was then conducted to further assess if these compositions were significantly different across collection sites. Using the *envfit* function from the *vegan* package, the four retained environmental parameters were fitted onto the PCA for each coral species to assess the correlation between these parameters and the compositions of Symbiodiniaceae DIV. Statistically significant environmental parameters in structuring the Symbiodiniaceae DIV compositions were then verified with PERMANOVA. The *betadisper* function was used to test for differences in within-group dispersions. The raw R code used for the analyses has been deposited in https://github.com/mingshengg/png_symbionts.

## Results

Of the 480 samples processed and sequenced, 413 with read counts exceeding 10,000 were retained (Table S4)*.* This resulted in an average per sample read count of 130,028 for *Diploastrea heliopora*, 166,819 for *Pachyseris speciosa*, 201,772 for *Pocillopora acuta*, and 141,485 for *Porites lutea*. All sequencing data have been deposited at the National Center for Biotechnology Information (BioProject ID: PRJNA977103).

**Fig. 2:**
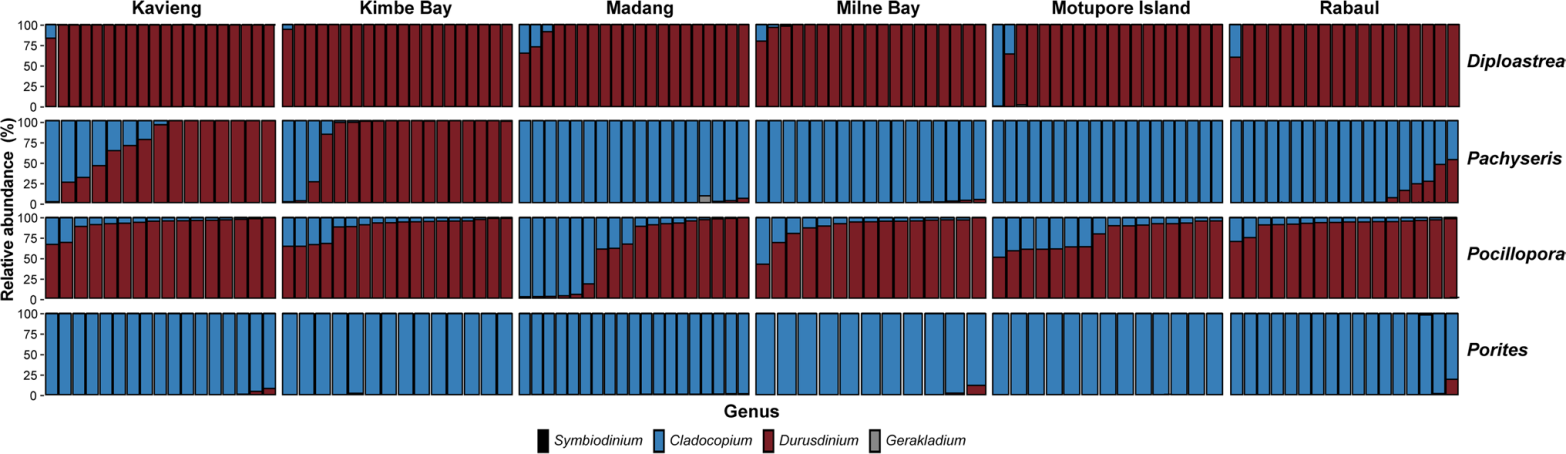
Symbiodiniaceae genera composition of the four coral species sampled from six sites across Papua New Guinea. Each stacked bar represents a single sample

The 413 coral samples collected from four coral species yielded a total of 1684 Symbiodiniaceae defining intragenomic variants (DIVs), resulting in 78 Symbiodiniaceae type profiles (set of co-occurring DIVs) from four genera, *Symbiodinium*, *Cladocopium*, *Durusdinium*, and *Gerakladium*. Specifically, there were 37 *Cladocopium*, 37 *Durusdinium*, 3 *Gerakladium*, and 1 *Symbiodinium* predicted type profiles. Notably, 34 of the 37 *Durusdinium* type profiles had D1 as the majority sequence, followed mostly by D4. Overall, *Durusdinium* and *Cladocopium* type profiles predominated in all 413 samples, constituting an average of 54.37 ± 2.29% (mean ± SE) and 45.60 ± 2.29% of the type profile compositions respectively (Fig. 2). Both *Pachyseris speciosa* and *Pocillopora acuta* were strongly associated with *Cladocopium* and *Durusdinium* type profiles. Specifically, *Pachyseris speciosa* hosted a higher proportion of *Cladocopium* (73.44 ± 3.98%) compared to *Durusdinium* profiles (26.48 ± 3.98%), while *Pocillopora acuta* hosted more *Durusdinium* (82.70 ± 2.39%) than *Cladocopium* profiles (17.29 ± 2.39%). *Diploastrea heliopora* comprised mostly of *Durusdinium* profiles (97.49 ± 1.04%) while *Porites lutea* was dominated almost entirely by *Cladocopium* profiles (99.39 ± 0.26%). While *Symbiodinium* DIVs were detected as background Symbiodiniaceae (< 1% abundance) in all coral species except *Diploastrea heliopora*, only one *Porites lutea* sample harbored *Symbiodinium* type profile at 1.58% relative abundance. Likewise, *Gerakladium* DIVs were also detected in the background of several samples across all four coral species but only three samples harbored *Gerakladium* type profiles. While *Fugacium* DIVs were detected, they were removed from subsequent analyses as they did not co-occur to form any type profiles, and were only detected in one sample at very low relative abundances, which suggests that they are unlikely to form stable associations with corals. *Fugacium* is also generally regarded as a free-living genus and a surface associate of corals rather than an endosymbiont [28, 56, 57], and has rarely been identified in other works across the Indo-Pacific region (e.g., absent in [58–60]). However, since rDNA copy number in *Cladocopium* can be five times more than *Durusdinium* [61], interpretation in the relative abundances of sequence reads especially in mixed endosymbiont communities with both *Cladocopium* and *Durusdinium* should be treated with due caution.

**Fig. 3:**
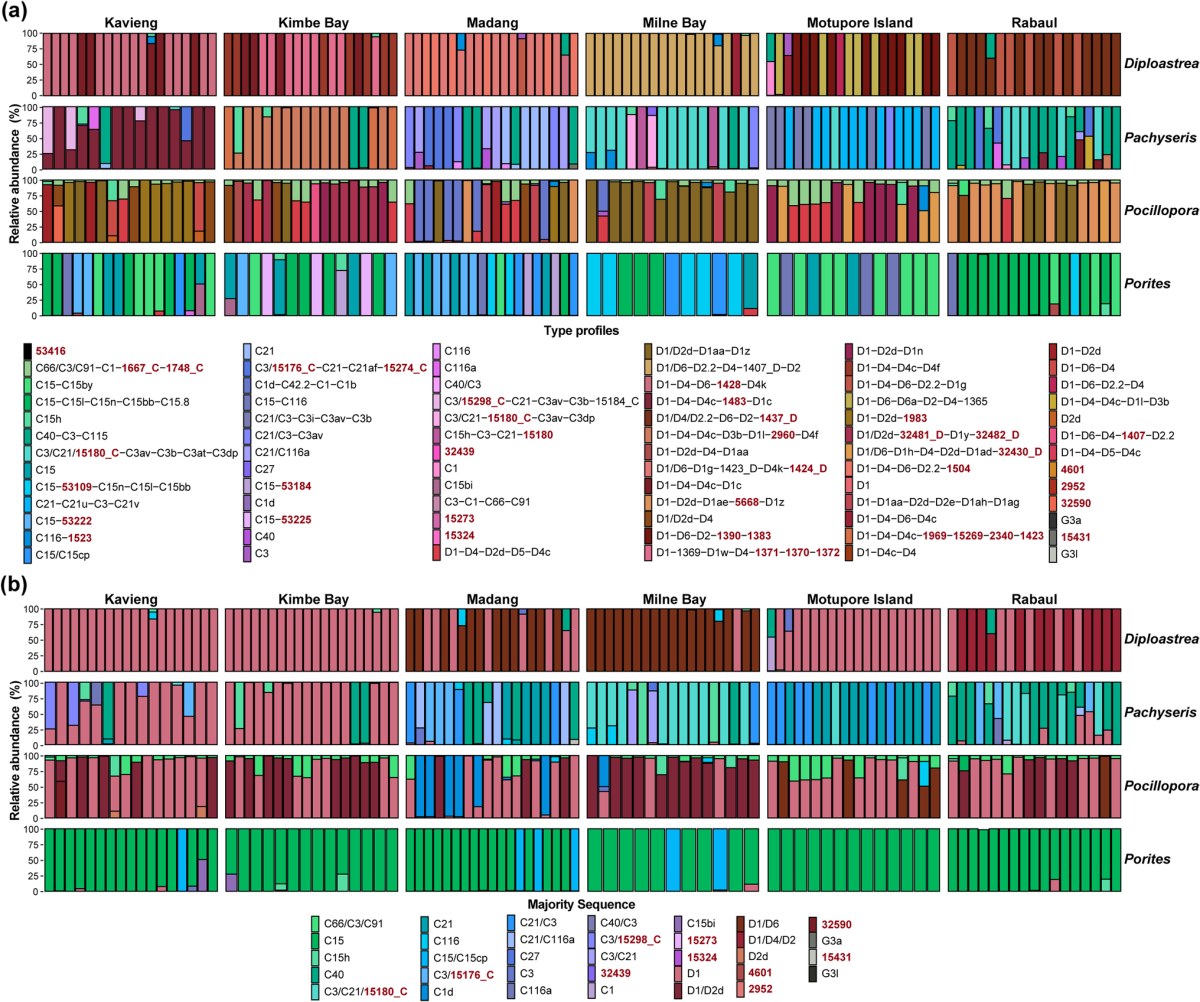
Composition of Symbiodiniaceae represented by the **a** type profiles and **b** the majority sequences of the type profiles of the four coral species sampled from six sites across Papua New Guinea. Each stacked bar represents a single sample. Cool colors (green to purple) represent *Cladocopium* profiles while warm colors (red to yellow) represent *Durusdinium* profiles. Type profiles and the majority sequences are arranged from most common to least common across samples. Defining intragenomic variants (DIVs) in red represent sequences not previously reported in the SymPortal database

Of the 78 identified type profiles, 33 contained DIVs that have not been deposited in the SymPortal database, with 10 type profiles having those as the majority sequence (Fig. 3). Sixty-one of the 78 type profiles were host-specific, with 30 of these further exclusively found at one sampling site (Fig. S3). For instance, the D1/D6-D2.2-D4_1407_D-D2 type profile was only found in *Diploastrea heliopora*, and was only identified in 18 samples from Milne Bay. Averaged across all sites, each coral sample hosted 1.53 ± 0.03 type profiles or 25.54 ± 0.53 DIVs. While 25 of the 1684 identified DIVs were present in at least one sample of each coral species (9 *Cladocopium* and 16 *Durusdinium* DIVs; Table S7), only the C15h type profile was found across all four coral species, occurring only in 22 samples.

The number of species-specific, site-specific, and genera distribution of type profiles of each coral species is summarized in Table 1. Notably, among the four studied coral species, *Pachyseris speciosa* exhibited the lowest symbiont specificity, hosting the greatest number of type profiles, and type profiles that were host- and site-specific (Table 1), whereas *Porites lutea* had the highest symbiont specificity with the least total and site-specific type profiles, with all 91 samples dominated by *Cladocopium* profiles with C15 as the majority sequence. Across the 99 *Pocillopora acuta* samples, there was an average of 2.14 ± 0.05 type profiles, with only three samples having one type profile, and the remaining 96 associated with both *Cladocopium* and *Durusdinium* type profiles.

**Table 1:** The number of Symbiodiniaceae type profiles across samples hosted by each coral species. Numbers in brackets indicate the number of species-specific type profiles that are further exclusive to a sampling site. Errors represent standard error

Host species	*Cladocopium* (Clade C)	*Durusdinium* (Clade D)	*Gerakladium* (Clade G)	Total	Species-specific type profiles	Type profiles per sample
*Diploastrea heliopora*	6	13	1	20	11 (9)	1.14 ± 0.04
*Pachyseris speciosa*	20	13	1	34	23 (12)	1.56 ± 0.06
*Pocillopora acuta*	9	16	0	25	16 (6)	2.14 ± 0.05
*Porites lutea*	13	4	1	18	11 (3)	1.32 ± 0.05

**Fig. 4:**
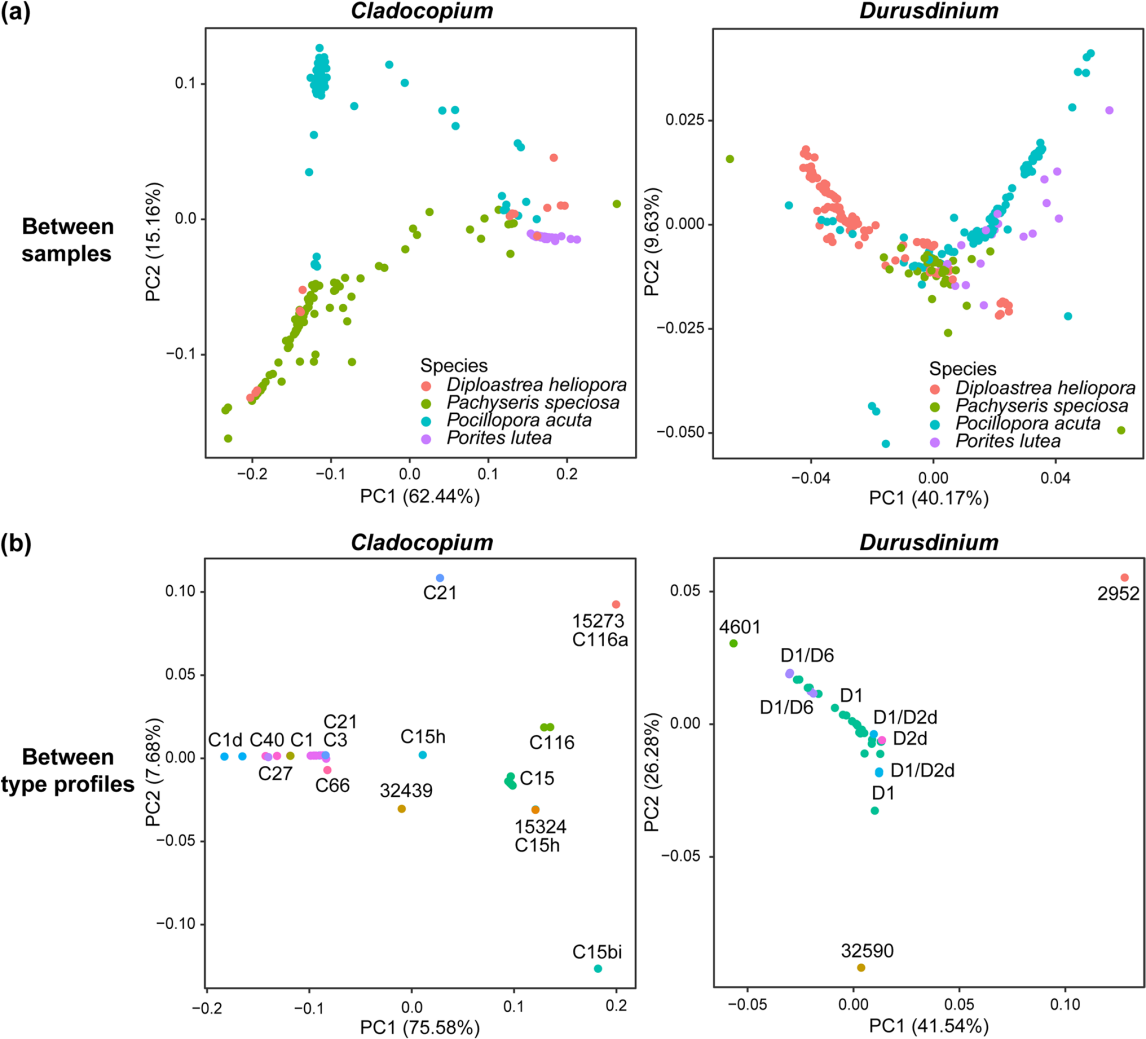
**a** Between-sample and **b** between-type profile principal coordinate analysis (PCoA) plots based on UniFrac distances of *Cladocopium* and *Durusdinium* ITS2 sequences. Points in (**a**) represent samples and are colored by coral species while points in (**b**) represent type profiles and are colored and labelled by their majority sequence(s)

Between-sample and between-ITS2 type profile principal coordinate (PCoA) plots were constructed to highlight host-specificity and intergenomic variation of *Cladocopium* and *Durusdinium* sequences (Fig. 4). The *Cladocopium* between-sample PCoA plot highlights that, generally, *Cladocopium* found in *Pachyseris speciosa*, *Pocillopora acuta*, and *Porites lutea* display host-specificity, with sequences from each coral species occupying distinct spaces on the PCoA plot (Fig. 4a). This was also exemplified with PERMANOVA as host species explained the most variation in the between-sample UniFrac distances (*R*^2^ value = 0.6378, *p*-value < 0.001, Table S5). Notably, *Porites lutea* displays a very high symbiont specificity, as indicated by the genotypes clustering tightly to occupy a small distinct space on the PCoA plot. In contrast, the *Cladocopium* genotypes found in *Pachyseris speciosa* and *Pocillopora acuta* show much higher intergenomic variation, with many genotypes having high levels of genetic similarity to the *Cladocopium* specific to other coral species, whereas *Cladocopium* sequences in *Diploastrea heliopora* samples did not display any host-specificity and overlapped either with those found in *Pachyseris speciosa* or *Porites lutea*. Likewise, the *Durusdinium* between-sample PCoA plot indicates that the predicted *Durusdinium* sequences also have considerable intergenomic variation and exhibit host-specificity, albeit to a much lower extent as genotypes are generally all clustered in the middle of the PCoA plot. This was also highlighted through PERMANOVA, where host species still explained the most variation but to a lesser extent when compared to the *Cladocopium* between-sample UniFrac distances (*R*^2^ value = 0.29839, *p*-value < 0.001).

The PCoA plot of *Cladocopium* sequences across type profiles highlights distinct groupings of type profiles with the same majority sequence (Fig. 4b). For instance, type profiles with majority sequence C15 have very high levels of genetic similarity and are clustered together to occupy a small space. Additionally, there were type profiles with different majority sequences with high genetic similarities, such as type profiles with C1, C3, C21, C27, C40, and C66 as their majority sequences. Conversely, *Durusdinium* type profiles, except for a few previously not reported sequences, are all clustered together with D1 as the majority sequence.

**Fig. 5:**
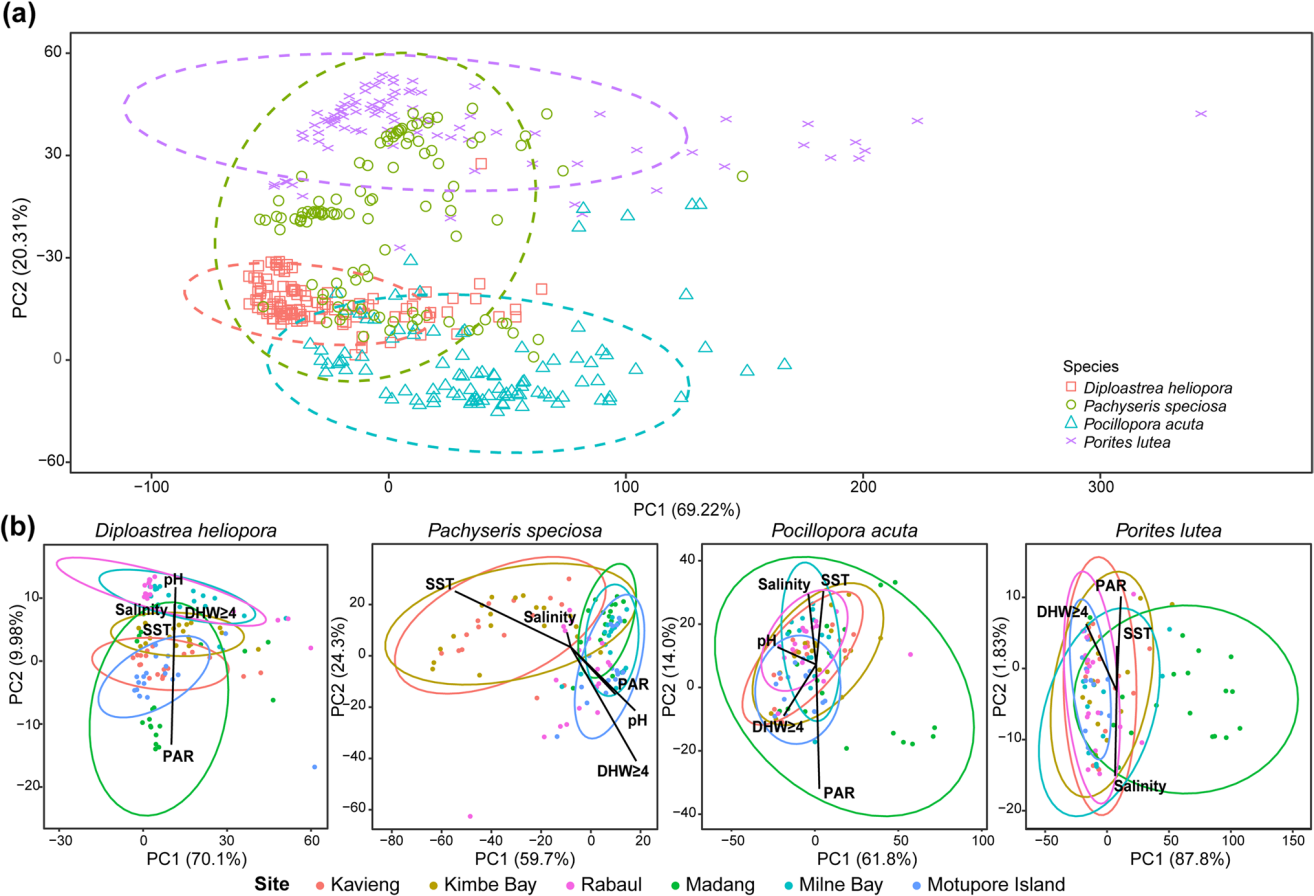
Principal component analysis (PCA) of Symbiodiniaceae type communities with robust Aitchison distances for **a** all four host species and **b** each host species with fitted vectors of environmental parameters (pH, DHW ≥ 4, PAR, SST, and salinity). Each point represents a coral sample from a particular site and ellipses denote 95% confidence level

Principal component analysis (PCA) and PERMANOVA were conducted to investigate whether Symbiodiniaceae DIV compositions differed significantly across coral species. The PCA plot of the 413 Symbiodiniaceae DIV compositions across all four coral host species had 69.22% of the variance explained by the principal component axis 1 (PC1) and 20.31% by the principal component axis 2 (PC2) (Fig. 5a). The 95% confidence ellipses showed that the compositions were generally distinct across coral species despite the overlapping regions. Further analysis via PERMANOVA with robust Aitchison distances revealed that the host species was the most significant driver of Symbiodiniaceae community structure, alongside the collection site and the interaction between species and collection site (Table 2).

**Table 2:** PERMANOVA (with 999 permutations) was conducted to investigate if coral host species, collection sites, and their interaction effects significantly affected the Symbiodiniaceae community compositions

	df	Sum Sqs	*R*^2^ value	Pseudo *F*	*p*-value
Species	3	8342	0.25548	55.7624	0.001
Site	5	1353	0.04143	5.4254	0.001
Species:site	15	3559	0.10900	4.7583	0.001
Residual	389	19399	0.59408		
Total	412	32653	1.00000		

Since the compositions of Symbiodiniaceae DIVs were species-specific, PCA and PERMANOVA were then conducted separately for each coral species to investigate site-specific patterns. The PCA illustrated slight site-specific clustering in Symbiodiniaceae DIV compositions (Fig. 5b), while PERMANOVA showed significant site-specific effects for all four coral species (Table S6). The compositions of DIVs in *Pachyseris speciosa* were the most dissimilar across sites (pseudo *F* statistic = 9.9317), followed by *Diploastrea heliopora* (pseudo *F* statistic = 5.9356), *Pocillopora acuta* (pseudo *F* statistic = 3.0647), and lastly *Porites lutea* (pseudo *F* statistic = 2.9279). The PC1 for *Pachyseris speciosa* also explained the least variance at 59.7% while the PC1 for *Porites lutea* explained the most variance at 87.8%. This illustrates that the composition of Symbiodiniaceae DIVs of *Pachyseris speciosa* had much greater variance across samples and sites compared to that of *Porites lutea*, contributing to the larger dispersion across-site as observed with PERMANOVA*.* However, within-site dispersions were heterogenous as demonstrated by *betadisper* in *Porites lutea* (*F* = 23.218, *p*-value < 0.001), *Pachyseris speciosa* (*F* = 6.4078, *p*-value < 0.001), and *Pocillopora acuta* (*F* = 6.9907, *p*-value < 0.001). Only *Diploastrea heliopora* had homogenous within-site dispersions (*F* = 0.4542, *p*-value = 0.810). While PERMANOVA may be affected by heterogeneity, especially for unbalanced designs [62], differences in sample sizes across sites for each coral species were small and their PCAs still showed relatively distinct structuring across sites, which suggests significant differences in Symbiodiniaceae compositions.

Results from a consensus approach with PERMANOVA and PCA showed that the Symbiodiniaceae compositions of the four corals were also shaped by environmental variables (Table 3). The number of degree heating week events reaching or exceeding 4 °C-weeks (DHW ≥ 4), sea surface temperature, photosynthetically available radiation (PAR), and salinity were significant in structuring the Symbiodiniaceae type profiles across all four coral species. The Symbiodiniaceae types of *Diploastrea heliopora*, *Pachyseris speciosa*, and *Pocillopora acuta* were further structured by pH levels. In particular, PAR was the most influential factor in structuring the Symbiodiniaceae compositions of *Diploastrea heliopora*, *Pocillopora acuta*, and *Porites lutea*, while DHW ≥ 4 was the most influential for *Pachyseris speciosa*.

**Table 3:** PERMANOVA pseudo F statistic for the five environmental parameters, number of degree heating week events reaching or exceeding 4 deg-C (DHW ≥ 4), photosynthetically available radiation (PAR), pH, sea surface temperature (SST), and salinity, in structuring Symbiodiniaceae type communities hosted by each coral species. Environmental parameters in bold were the strongest in shaping the Symbiodiniaceae type communities for each species. Stars indicate significant levels: *p-value < 0.05, **p-value < 0.01, and ***p-value < 0.001

Species	Pseudo *F* statistic				
*Diploastrea heliopora*	DHW ≥ 4 3.5782 ***	**PAR** 10.3675 ***	pH 3.7088 ***	SST 5.4409 ***	Salinity 6.5827 ***
*Pachyseris speciosa*	**DHW** ≥ **4** 21.5258 ***	PAR 2.8172 *	pH 4.7070 ***	SST 15.0364 ***	Salinity 4.6157 **
*Pocillopora acuta*	DHW ≥ 4 1.9135 *	**PAR** 4.5806 ***	pH 2.0907 *	SST 3.3453 **	Salinity 3.2071 ***
*Porites lutea*	DHW ≥ 4 2.7433 **	**PAR** **5.0035** ***		SST 3.0578 **	Salinity 2.7015 **

## Discussion

Consistent with other reef systems in the region, the corals in Papua New Guinea (PNG) are dominated by *Cladocopium* and *Durusdinium*, but demonstrate significant intra- and intergenomic variation. Across the 413 individuals from four coral species, 1684 defining intragenomic variants (DIVs) and 78 type profiles were identified, with 33 of these containing previously unreported DIVs. This prominent diversity largely stems from a very high host- and site-specificity, with 61 type profiles exclusive to a coral species, and 30 of these further exclusive to a sampling site. In addition to host- and site-specific effects, environmental factors also contribute to the structuring of the symbiont partners in coral hosts. In particular, photosynthetically active radiation (PAR) emerged as the most influential environmental variable in structuring the Symbiodiniaceae in *Diploastrea heliopora*, *Pocillopora acuta*, and *Porites lutea*, while the number of thermal anomaly events that can cause bleaching (measured as number of degree heating weeks, DHW, reaching or exceeding 4 °C-weeks) was the most influential for *Pachyseris speciosa*. Altogether, this work represents the first profiling of the Symbiodiniaceae communities of corals across PNG and provides evidence that the Symbiodiniaceae harbored by the corals here are remarkably diverse in comparison to other sites within the region.

The 413 coral fragments from four coral species sampled across six reef sites in PNG identified four symbiont genera, *Cladocopium*, *Durusdinium*, *Gerakladium*, and *Symbiodinium*. Consistent with other Indo-Pacific reef systems [21, 58, 59, 63], *Cladocopium* and *Durusdinium* endosymbionts are dominant in PNG corals. Meanwhile, *Symbiodinium* and *Gerakladium* defining intragenomic variants (DIVs) were present at low background levels across several coral samples, although only *Gerakladium* type profiles were identified by SymPortal. Similar observations of *Gerakladium* and *Symbiodinium* at low background levels have been reported in corals of reef systems across the Coral Triangle such as Malaysia and Singapore [21, 64]. The dominance patterns of *Cladocopium* and *Durusdinium* were also consistent with previous research on the same coral species, particularly the dominance of *Durusdinium* in *Diploastrea heliopora* and *Cladocopium* in *Porites lutea* [21, 58, 59, 65, 66]*,* indicating a pronounced preference for specific Symbiodiniaceae and highlighting the role of the coral host in structuring its endosymbiont community.

This work identified a total of 1684 unique Symbiodiniaceae DIVs and 77 type profiles across 413 samples collected from four species of coral, far surpassing the numbers reported in prior studies within the Asia–Pacific region. For example, 263 samples across three coral species in Singapore identified only 15 type profiles [59], while 60 samples across 30 different coral species from Hong Kong only identified 13 type profiles [67]. While this study had a broader sampling range, a recurring observation in coral-Symbiodiniaceae communities across the region is the prevalence of certain type profiles across coral hosts and/or sampling sites [68–70]. A clear illustrative example is the *Cladocopium* type profile C3u/C3/C115/C27-C3bn; this profile was found consistently across all six sampling sites and five coral species in research performed in Singapore [71]. In contrast, the type profiles identified in PNG exhibited marked host- and site-specificity. For instance, among the 11 type profiles unique to *Diploastrea heliopora*, nine were further exclusive to a specific sampling site where they were dominant within their host. Illustratively, the type profile D1/D6-D2.2-D4-1407_D-D2 was only found in 18 *Diploastrea heliopora* samples from Milne Bay at an average relative abundance of 98.62 ± 1.11%. This pattern of host- and site-specificity contributes to the high diversity of type profiles in PNG corals.

Type profiles are generally considered to represent distinct genotypes of Symbiodiniaceae and are indicative of putative taxa [47]. However, the tight clustering of type profiles, especially those with the same majority sequence in the principal coordinate analysis (PCoA) plots suggests that each cluster likely represents groups of closely related strains instead of distinct species. For example, almost all *Durusdinium* type profiles formed a tight cluster on the PCoA plot with D1 as the majority sequence followed by D4 sequences, indicative of *D. trenchii* [72]. This suggests that these *Durusdinium* type profiles represent closely related strains of *D. trenchii* rather than putative taxa. Likewise, many of the *Cladocopium* type profiles with C1 or C40 as their majority sequences likely represent the recently described *C. vulgare* and *C. madreporum*, respectively, common across many species of corals throughout the Indo-Pacific [73]. Regardless, these type profiles exhibited marked intergenomic variation across distinct hosts and sampling sites to be characterized as distinct type profiles. Similar patterns of high intergenomic variation and host species-specific genotypes in *D. trenchii* were also observed in Red Sea corals [74].

In addition to the high number of type profiles identified here, the number of DIVs hosted by each coral sample was also far greater than in other studies. For example, *Pocillopora acuta* samples in our study harbored an average of 36.49 ± 0.66 DIVs, while those collected across Malaysia and Singapore had 10.8 to 24.2 DIVs [21, 64]. Since each DIV represents an ITS2 intragenomic variant, this underscores the greater intragenomic sequence diversity within the corals of PNG compared to other Indo-Pacific reef systems. This extensive intra- and intergenomic variation, coupled with many previously unreported sequences, suggests that coral-Symbiodiniaceae diversity in PNG surpasses that of other reef systems across the region. Reefs considered resilient often harbor homogenous Symbiodiniaceae communities with low diversities, a consequence of these communities shifting towards stress-tolerant taxa with each bleaching event [24, 75, 76]. Despite being situated in the West Pacific Warm Pool and having experienced multiple bleaching events [4, 8], the corals in PNG have seemingly evaded the typical Symbiodiniaceae community restructuring and instead persist in maintaining endosymbiont communities with high intra- and intergenomic variations. However, these endosymbiont restructuring events are crucial for enhancing the corals’ ability to thrive in high temperature conditions [77]. The observed diversity in PNG could therefore potentially render its reefs more susceptible to future bleaching events, especially if they become more severe and frequent [38]. On the contrary, coral-Symbiodinium symbioses can become less stable with each restructuring event, and the high genetic and community-level variation present in PNG may help the corals persist in changing climates [78–80]. In all cases, this emphasizes the need for greater protection and more research in hotspots with apparent high symbiont diversity like PNG.

Expectedly, the structure of Symbiodiniaceae partners was largely determined by the coral host, with sampling sites playing a comparatively minor role. This was substantiated by the concordance between the variance allocated in the PERMANOVA results, the host-specific clustering of the predicted genotypes especially in *Cladocopium*, and the low number of type profiles shared across coral hosts. Between-sample PCoA plots show that *Cladocopium* genotypes from one coral host can display intermediate to very high levels of genetic similarity with those from another coral host, despite the generally pronounced host-specificity. These *Cladocopium* may have originated from nearby corals of different species [81], especially since spawning corals, like those studied here, primarily acquire their algal endosymbionts horizontally, which are then selectively regulated by the host to give rise to the observed specificity [82, 83]. Alternatively, these sequences may represent hybrids between endosymbionts specific to two distinct coral hosts, considering the growing evidence of sexual reproduction within Symbiodiniaceae [84–86]. Whether these genotypes represent distinct species or hybrids will necessitate the use of additional markers, but these novel genotypes could represent physiologically advantageous symbionts that have adapted to their specific environments [87, 88]. Whereas *Diploastrea heliopora* were largely associated with *Durusdinium* symbionts, its background *Cladocopium* sequences overlapped with those of *Porites lutea* and *Pachyseris speciosa*, suggesting that these *Cladocopium* symbionts are host-generalists and associate across different coral hosts [73].

Although the number of DHW ≥ 4 events was significant in shaping the Symbiodiniaceae of all four examined coral species, it was the most influential variable only in *Pachyseris speciosa*. Degree heating weeks (DHW) is defined as the accumulation of temperature anomalies exceeding the monthly maximum mean sea surface temperature [89]. Symbiont cell morphology and cell division rates can start to alter at 2 °C-weeks [90], transcriptional changes in Symbiodiniaceae can occur at 3 °C-weeks [91], while 4 °C-weeks and beyond is associated with ecologically significant bleaching levels and breakdown of coral assemblage structure [89, 92, 93]. In fact, DHW consistently reached or exceeded 8 °C-weeks in PNG, which is thought to result in reef-wide bleaching and coral mortalities [94]. Yet, these bleaching and symbiont restructuring events were only the most significant in *Pachyseris speciosa*, and combined with the still high intra- and intergenomic Symbiodiniaceae diversity in PNG, seems to suggest that the thermal anomaly events did not result in severe recurring bleaching events and the symbiont community homogenization that follows [19, 76, 95]. Whether the high symbiont diversity is conferring thermal resilience to the corals requires a more thorough investigation, but without long-term investigation of local stressors, it is difficult to predict in situ bleaching events and if or how the reef-wide symbiont communities are restructured [96, 97].

Algal symbionts of the other three coral species were largely structured by PAR. Specific symbionts can thrive in distinct photic environments [98], with specific Symbiodiniaceae thriving in high-light environments, while others prefer darker conditions [99]. High light exposure can also synergistically cause bleaching with heat stress, exacerbating bleaching intensity [100] and accelerating shifts in Symbiodiniaceae [101]. Culture-based experiments have further demonstrated how different Symbiodiniaceae members have varying tolerance to thermal and light stress [102], which when coupled with host-specific interactions could have marked effects on coral physiological performance [103]. Nevertheless, the within-group distributions of *Pachyseris speciosa*, *Pocillopora acuta*, and *Porites lutea* were also highly significant, suggesting intraspecific variation [63] and/or fine-scale environmental differences such as depth [104] also played a significant role in shaping the coral’s endosymbiont partners even within the same sites. For a more comprehensive investigation of Symbiodiniaceae structuring and assembly, future studies should combine large-scale datasets such as thermal anomalies, light levels, and upwelling [105], with fine-scale variables such as reef structure, depth, and turbidity [106, 107] to monitor long-term temporal and spatial stability of these Symbiodiniaceae communities.

In conclusion, our study sheds light on the diverse Symbiodiniaceae communities associated with corals in PNG. We found that the Symbiodiniaceae communities here were broadly similar to neighboring regions such as the Great Barrier Reef and the wider Coral Triangle. However, PNG reefs hosted Symbiodiniaceae communities with much greater intra- and intergenomic variation compared to other reef systems across the Indo-Pacific region, suggesting the possibility of PNG as a hotspot of coral endosymbiont diversity. The genera *Cladocopium* and *Durusdinium* emerged as the dominant symbionts, with their dominance patterns mainly influenced by host-specificity and site-specific environmental factors. Coral-Symbiodiniaceae compositions were further shaped by the unique suite of environmental variables present at each sampling site. The reefs of PNG and the associated warm water masses of the West Pacific Warm Pool could offer a glimpse into how the reefs of the Coral Triangle will adapt to ongoing climate warming.

### Supplementary Information

Below is the link to the electronic supplementary material.

Supplementary file1 (DOCX 837 KB)

## Data Availability

All sequences associated with this work have been deposited at the National Center for Biotechnology Information under BioProject ID: PRJNA977103. Codes used for the analyses are available on GitHub, https://github.com/mingshengg/png_symbionts.
