# Supplementary material for: Highly Diverse Symbiodiniaceae Types Hosted by Corals in a Global Hotspot of Marine Biodiversity
Source: Microb Ecol. 2024 Jul 10;87(1):92. doi: 10.1007/s00248-024-02407-x (PMC11236936; doi:10.1007/s00248-024-02407-x)
Supplement: Supplementary file 1 — Supplementary file1 (DOCX 837 KB) [file 248_2024_2407_MOESM1_ESM.docx]

**Highly diverse Symbiodiniaceae communities hosted by corals in a global hotspot of marine biodiversity**

Ming Sheng Ng^1^, Nathaniel Soon^2^, Lutfi Afiq-Rosli^3^, Ismael Kunning^4^, Ralph R. Mana^4^, Ying Chang^1,2^, Benjamin J. Wainwright^1,2^

^1^ Department of Biological Sciences, National University of Singapore, Singapore

^2^ Yale-NUS College, National University of Singapore, Singapore

^3^ Red Sea Research Center, Biological and Environmental Sciences and Engineering Division (BESE), King Abdullah University of Science and Technology (KAUST), Thuwal, Saudi Arabia

^4^ School of Natural and Physical Sciences, University of Papua New Guinea, Port Moresby, Papua New Guinea

*Corresponding Author: Benjamin J. Wainwright, Yale-NUS College, National University of Singapore, 16 College Avenue West, Singapore 138527, Singapore

Email: [Ben.Wainwright@Yale-NUS.edu.sg](mailto:Ben.Wainwright@Yale-NUS.edu.sg)

**Table S1** Adapters and primers used for Symbiodiniaceae ITS2 amplification, from Hume et al. (2018). Primers and PCR thermal cycling conditions used in study. Underlined is the gene primer, the remainder is overhang and is used to attach unique barcodes and flowcell adaptors to PCR products in the second round PCR. In the index primer used in the 2nd PCR, [i5] & [i7] represent unique barcodes used in demultiplexing, the sequence preceding this is the Illumina adaptor and the sequence after this allows attachment to the overhang on the gene primer.

| Symbiodiniaceae  ITS2 Gene PCR | Forward: 5’ – TCGTCGGCAGCGTCAGATGTGTATAAGAGACAGGAATTGCAGAACTCCGTGAACC -3’  Reverse: 5’ – GTCTCGTGGGCTCGGAGATGTGTATAAGAGACAGCGGGTTCWCTTGTYTGACTTCATGC – 3’ |
| --- | --- |
|  | 1 × 95 °C for 3 min  25 × 95 °C for 30 sec, 55 °C for 30 sec, 72 °C for 30 sec  1 × 72 °C for 5 min |
| Symbiodiniaceae  ITS2 Index PCR | Forward: 5’–AATGATACGGCGACCACCGAGATCTACAC[i5]TCGTCGGCAGCGTC –3’  Reverse: 5’– CAAGCAGAAGACGGCATACGAGAT[i7]GTCTCGTGGGCTCGG –3’ |
|  | 1 × 95 °C for 3 min  8 × 95 °C for 30 sec, 55 °C for 30 sec, 72 °C for 30 sec  1 × 72 °C for 5 min |

**Table S2** Five of the fourteen parameters retained from Bio-ORACLE v2.1 (Tyberghein et al., 2012; Assis et al., 2018) after removal of collinear parameters. Data was extracted from the latest version available for the parameter.

| Parameter | Version | Time period | Layer ID | Description |
| --- | --- | --- | --- | --- |
| Sea surface temperature | 1.0 | 2002 – 2009 | 1066 | Mean sea surface temperature |
| Photosynthetically available radiation | 1.0 | 1997-2009 | 460 | Mean photosynthetically available radiation |
| pH | 2.1 | 1910 - 2007 | 461 | pH; measure of acidity in the ocean |
| Salinity | 2.1 | 2000 – 2014 | 1008 | Mean sea surface salinity |

**Table S3** Long-term averaged sea surface environmental data extracted from Bio-ORACLE v2.1 (Tyberghein et al., 2012; Assis et al., 2018) and the number of degree heating week events from 1985 – 2023 extracted from NOAA Coral Reef Watch. Highest and lowest values for each environmental parameter across sites are highlighted in orange and blue respectively.

| Parameter | Kavieng | Rabaul | Kimbe Bay | Madang | Milne Bay | Motupore Island |
| --- | --- | --- | --- | --- | --- | --- |
| Sea surface temperature (°C) | 29.79 | 29.56 | 29.74 | 28.90 | 27.93 | 27.49 |
| Photosynthetically available radiation (E m^-2^ d^-1^) | 43.18 | 44.63 | 44.60 | 45.69 | 42.46 | 47.50 |
| pH | 8.276 | 8.293 | 8.299 | 8.269 | 8.362 | 8.35 |
| Salinity (PSS) | 34.48 | 34.23 | 34.04 | 34.18 | 34.44 | 34.08 |
| Number of Degree Heating Week events ≥ 4°C-weeks | 3 | 8 | 5 | 4 | 7 | 11 |

**Table S4** Number of samples per species from each site after sequence processing and filtering.

| Type | Site | Coordinates | *Diploastrea heliopora* | *Pocillopora acuta* | *Pachyseris speciosa* | *Porites lutea* |
| --- | --- | --- | --- | --- | --- | --- |
| Archipelago | Kavieng | -2.573474°N  150.795224°E | 20 | 18 | 18 | 14 |
| Archipelago | Rabaul | -4.198459°N  152.172829°E | 18 | 16 | 19 | 17 |
| Archipelago | Kimbe Bay | -5.550205°N  150.144544°E | 20 | 16 | 15 | 17 |
| Mainland | Madang | -4.896403°N  145.765363°E | 20 | 18 | 18 | 19 |
| Mainland | Milne Bay | -10.022649°N  151.580029°E | 19 | 15 | 17 | 11 |
| Mainland | Motupore Island | -9.523889°N  147.284722°E | 20 | 16 | 19 | 13 |
|  | **Total** |  | **117** | **99** | **106** | **91** |

**Table S5.** PERMANOVA (with 999 permutations) was conducted on the between-sample UniFrac distances to investigate if coral host, collection site, and their interaction has a significant effect on the structuring of Symbiodiniaceae type profiles in corals.

| Symbiodiniaceae | Variable | df | Sum Sqs | R^2^ value | Pseudo F | *p-*value |
| --- | --- | --- | --- | --- | --- | --- |
| *Cladocopium* | Species | 3 | 0.066798 | 0.63748 | 223.3955 | 0.001 |
|  | Site | 5 | 0.001874 | 0.01789 | 3.7609 | 0.001 |
|  | Species:Site | 15 | 0.005513 | 0.05261 | 3.6873 | 0.001 |
|  | Residual | 307 | 0.030599 | 0.29202 |  |  |
|  | Total | 330 | 0.104784 | 1.00000 |  |  |
| *Durusdinium* | Species | 3 | 0.0012101 | 0.29838 | 63.5142 | 0.001 |
|  | Site | 5 | 0.0003595 | 0.08864 | 11.3208 | 0.001 |
|  | Species:Site | 15 | 0.0007014 | 0.17294 | 7.3626 | 0.001 |
|  | Residual | 281 | 0.0017846 | 0.44003 |  |  |
|  | Total | 304 | 0.0040557 | 1.00000 |  |  |

**Table S6** PERMANOVA (with 999 permutations) was conducted on each coral host species separately to investigate if collection site has a significant effect on the composition of their Symbiodiniaceae DIV compositions.

| Species | Variable | df | Sum Sqs | R^2^ value | Pseudo F | *p-*value |
| --- | --- | --- | --- | --- | --- | --- |
| *Diploastrea heliopora* | Collection site | 5 | 755.3 | 0.21096 | 5.9356 | 0.001 |
|  | Residual | 111 | 2825.1 | 0.78904 |  |  |
|  | Total | 116 | 3580.4 | 1.00000 |  |  |
| *Pachyseris speciosa* | Collection site | 5 | 2132.3 | 0.33181 | 9.9317 | 0.001 |
|  | Residual | 100 | 4294.0 | 0.66819 |  |  |
|  | Total | 105 | 6426.3 | 1.00000 |  |  |
| *Pocillopora acuta* | Collection site | 5 | 1095.8 | 0.14146 | 3.0647 | 0.001 |
|  | Residual | 93 | 6650.4 | 0.85854 |  |  |
|  | Total | 98 | 7746.1 | 1.00000 |  |  |
| *Porites lutea* | Collection site | 5 | 874.4 | 0.14841 | 2.9279 | 0.001 |
|  | Residual | 84 | 5017.1 | 0.85159 |  |  |
|  | Total | 89 | 5891.5 | 1.00000 |  |  |

**Table S7** The 25 Symbiodiniaceae ITS2 DIVs shared found in at least one sample across the four species.

| Genus | ITS2 DIVs |
| --- | --- |
| *Cladocopium* | C3, C15, C15h, C15do, C116, C116a, 1514_C, 1523_C, 1527_C |
| *Durusdinium* | D1, D1b, D1c, D1h, D1l, D1r, D1n, D1ad, D2, D2a, D4, D4c, D4f, D6, 1437_D, 1637_D |


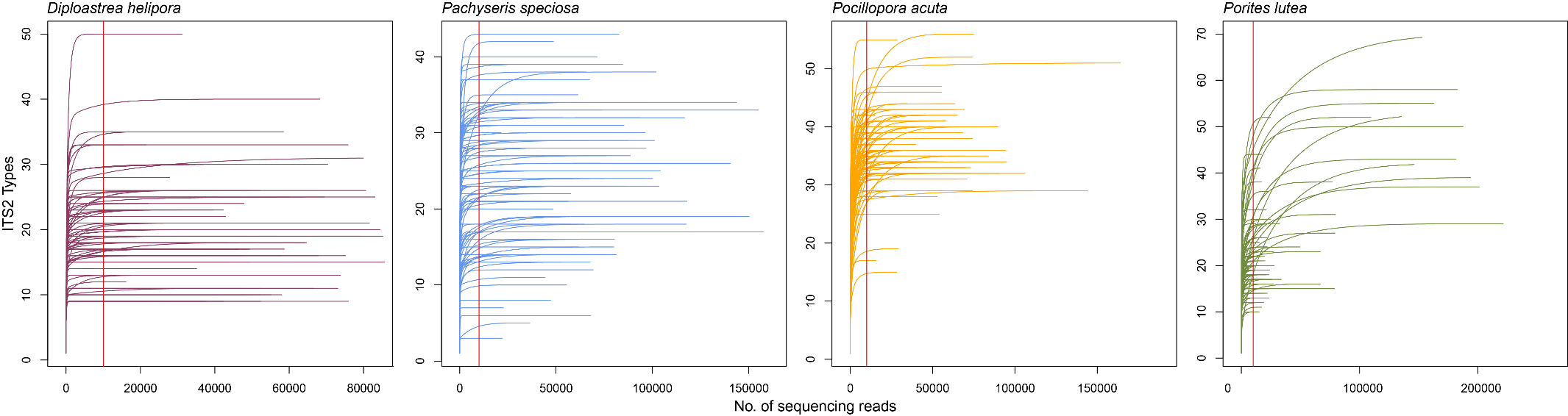


**Fig. S1** Rarefaction curves of samples for each coral species, all with at least 10,000 sequencing reads (vertical red line).


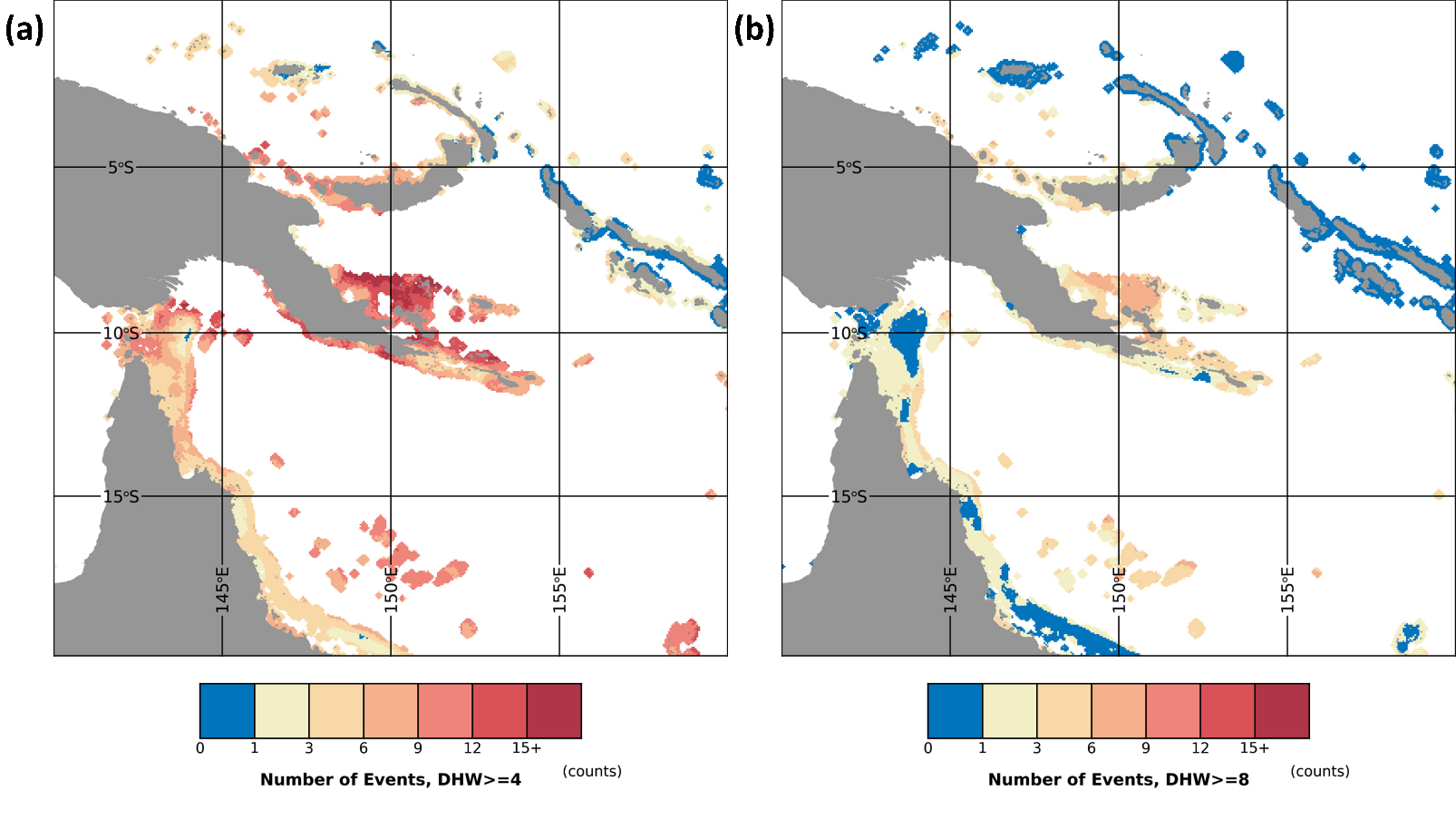


**Fig. S2** Number of events where DHW reached or exceeded (a) four and (b) eight between 1985 and 2023. Images are taken from NOAA Coral Reef Watch (Thermal History – Stress Frequency, Version 3.5) from <https://coralreefwatch.noaa.gov/product/thermal_history/stress_frequency.php>.


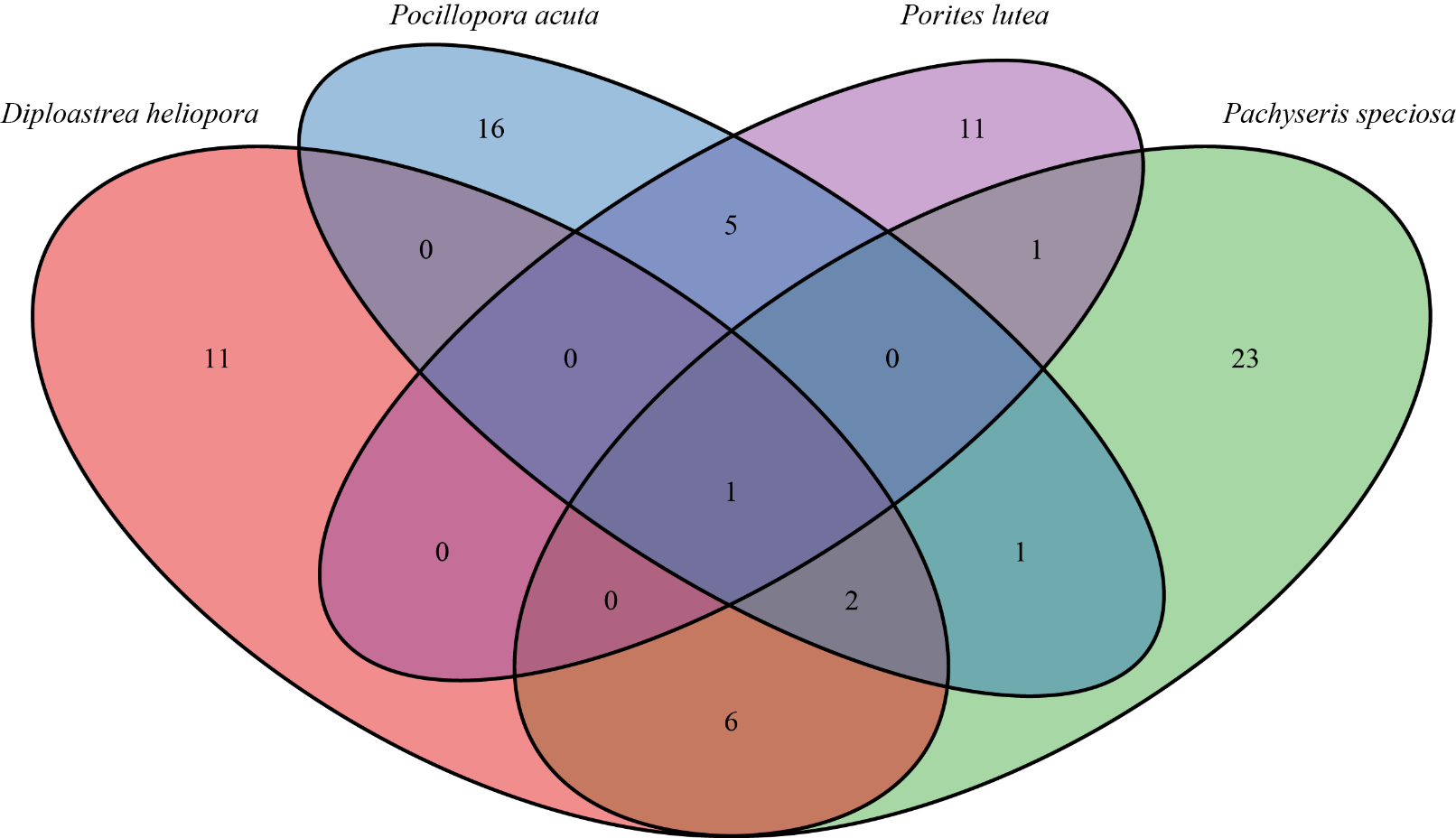


**Fig. S3** Number of ITS2 type profiles shared across the four coral host species.
